# Accurate Quantitative Sensing of Intracellular pH based on Self-ratiometric Upconversion Luminescent Nanoprobe

**DOI:** 10.1038/srep38617

**Published:** 2016-12-09

**Authors:** Cuixia Li, Jing Zuo, Li Zhang, Yulei Chang, Youlin Zhang, Langping Tu, Xiaomin Liu, Bin Xue, Qiqing Li, Huiying Zhao, Hong Zhang, Xianggui Kong

**Affiliations:** 1 State Key Laboratory of Luminescence and Applications, Changchun Institute of Optics, Fine Mechanics and Physics, Chinese Academy of Sciences, Changchun 130033, China; 2 Graduate University of the Chinese Academy of Sciences, Beijing 100049, China; 3 Van’t Hoff Institute for Molecular Sciences, University of Amsterdam, Science Park 904, 1098 XH Amsterdam, The Netherlands; 4 Department of Basic Medicine, Gerontology Department of First Bethune Hospital, University of Jilin, Changchun 130021, China

## Abstract

Accurate quantitation of intracellular pH (pH_i_) is of great importance in revealing the cellular activities and early warning of diseases. A series of fluorescence-based nano-bioprobes composed of different nanoparticles or/and dye pairs have already been developed for pH_i_ sensing. Till now, biological auto-fluorescence background upon UV-Vis excitation and severe photo-bleaching of dyes are the two main factors impeding the accurate quantitative detection of pH_i_. Herein, we have developed a self-ratiometric luminescence nanoprobe based on förster resonant energy transfer (FRET) for probing pH_i_, in which pH-sensitive fluorescein isothiocyanate (FITC) and upconversion nanoparticles (UCNPs) were served as energy acceptor and donor, respectively. Under 980 nm excitation, upconversion emission bands at 475 nm and 645 nm of NaYF_4_:Yb^3+^, Tm^3+^ UCNPs were used as pH_i_ response and self-ratiometric reference signal, respectively. This direct quantitative sensing approach has circumvented the traditional software-based subsequent processing of images which may lead to relatively large uncertainty of the results. Due to efficient FRET and fluorescence background free, a highly-sensitive and accurate sensing has been achieved, featured by 3.56 per unit change in pH_i_ value 3.0–7.0 with deviation less than 0.43. This approach shall facilitate the researches in pH_i_ related areas and development of the intracellular drug delivery systems.

pH_i_ plays a pivotal role in the modulation of cellular behaviors, including cell metabolism, proliferation, apoptosis, as well as vesicle trafficking *etc*. Abnormal (Acidic) pH_i_ may symbolize the dysfunctions of cells and diseases, such as cancer, Alzheimer’s *etc*^1^,^2^,^3^,^4^. Highly-sensitive sensing and accurate quantitative measurement of pH_i_ are thus greatly desired in the field of molecular biology and medicine research^5^,^6^,^7^. Till now, various fluorescence-based approaches of pH_i_ detection have been developed^8^,^9^,^10^,^11^,^12^. Especially in recent years the ratiometric fluorescent sensing strategies which depend on two fluorescence signals have been widely adopted. These strategies can minimize the influence of measurement condition changes (such as fluctuating light sources, probe concentration and optical path *etc.*)^3^,^5^,^8^,^9^,^10^,^13^,^14^,^15^. Various nanomaterials are used to develop ratiometric sensing nanoprobes, such as latex, polymer, silica, QDs and polymer dots *etc.* as matrix with loading different dyes (usually pH-sensitive and pH-insensitive)^3^,^4^,^5^,^6^,^16^,^17^,^18^,^19^,^20^. The drawback of these nanoprobes comes from the request of ultraviolet (UV) or visible (Vis) light excitation, which may lead to certain inevitable side-effects, such as strong disruptive auto-fluorescence background in biological samples, serious cellular photo damage and dyes photo-bleaching *etc*. Moreover, two separated signal measurements are always needed for ratiometric detection, where the change in light sources and filter sets may result in time-shifted recording or, even worse, the change of the measurement geometry^11^. In addition, the software-based subsequent processing (such as MATLAB, Olympus software *etc.*) is essential in most current quantification approaches, where the possible presence of large deviation is also a critical concern^3^,^5^,^9^. Therefore, it is an exceeding desire of developing a novel nanoprobe which can circumvent these problems and realize a reliable, sensitive and accurate pH_i_ measurement.

UCNPs have unique advantages for bioapplications. Typically, they can be excited by near infrared (NIR) light which avoids biofluorescence background^21^,^22^,^23^. In addition, UCNPs have large anti-stokes emissions (>300 nm), good photo-stability, lower cytotoxicity and narrow multi-emission bands *etc.*, which are also advantageous to bio-applications in complex biological system, such as whole blood, cell, tissues *etc*^24^,^25^,^26^,^27^. Despite the intensive exploration of UCNPs in bio-sensing and bio-imaging, employing UCNPs for intracellular pH sensing is still limited^28^,^29^,^30^. And reasons include (1) the low FRET efficiency, due to the large distance (in the case of thick silica shell) or the poor spectral overlap between UCNPs (energy donor, D) and pH indicators (energy acceptor, A), severely hampers the detection sensitivity and accuracy, and (2) the sensitivity of pH indicators loaded on UCNPs is a serious concern. Consequently, Employing UCNPs in quantitative pH_i_ sensing is still a great challenge. As far as pH indicators are concerned, FITC is popular due to its high sensitivity to pH^13^,^31^,^32^,^33^. However, the photo-bleaching under UV-Vis excitation and the easy release of FITC from cells have hampered its application in pH_i_ sensing, especially in quantitative pH_i_ measurement.

Here, we have developed a highly-sensitive self-ratiometric nanoprobe integrating NaYF_4_:Yb^3+^, Tm^3+^ UCNPs and FITC for accurate quantitative pH_i_ sensing, in which the absorption band of FITC matches better with the upconversion emission band at 475 nm. The intensity of upconversion emission at 475 nm (*I*_*475*_) and the intensity at 645 nm (*I*_*645*_) are used as the response signal and the reference signal, respectively. The as-prepared nanoprobe effectively eliminated photo-bleaching of FITC in pH_i_ measurement due to no UV-Vis light excitation of FITC. Under NIR-excitation without the undesired fluorescence background and photo damage, an accurate quantitative measurement has been realized using the self-ratiometric nanoprobe, avoiding the popularly used imaging-based subsequent software processing which may bring in large deviation.

## Results and Discussion

### Principle of F-UCNPs-based self-ratiometric quantitative pH_i_ sensing

**Figure 1:**
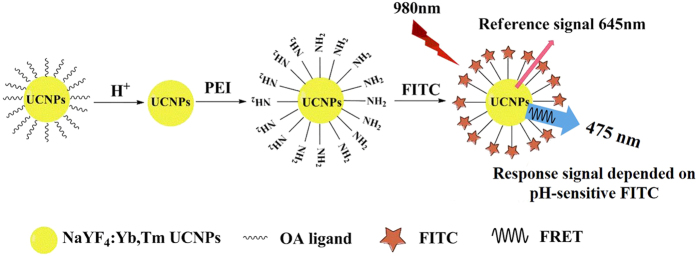
Schematic construction of F-UCNPs nanoprobe for self-ratiometric pH sensing.

Taking advantage of the properties of narrow multi-emission bands of UCNPs and a large spectral overlap between the absorption of FITC and the emission at 475 nm of UCNPs, F-UCNPs nanoprobe was designed to realize the self-ratiometric pH sensing with UCNPs as donors and FITC as acceptors, as shown in Fig. 1. FITC as pH indicator was conjugated on the surface of PEI-UCNPs by the PEI ligand. This design can avoid the embedded dyes leaking from the matrices which has been a big concern for nanoparticle-based pH sensors^8^,^33^,^34^. Upon 980 nm excitation, two upconversion luminescence bands appear at 475 nm and 645 nm, respectively. The intensity of the one at 475 nm, which was subject to the energy transfer between UCNPs and FITC, was used as response signal. The upconversion emission at 645 nm is inert to pH variation and used as reference signal, because energy transfer will not occur between UCNPs and FITC due to no spectra overlap between the two. A self-ratiometric measurement can be realized by monitoring the ratio (*I*_475/645_) of the upconversion luminescence intensities *I*_475_ and *I*_645_. As a result, the influence of variation of the measurement condition on the detection accuracy can be minimized. In addition, the pH-responsive fluorescence signal and the self-ratiometric reference signal are all derived from UCNPs, which avoids FITC loading limitation resulting from self-quenching effect. In turn, the high FITC loading is favorable for efficient energy transfer. Therefore, the UCNPs-based self-ratiometric sensing approach may lead to a highly sensitive and accurate quantitative measurement of pH_i_.

### Characterization of NaYF_4_:Yb^3+^, Tm^3+^ UCNPs

**Figure 2:**
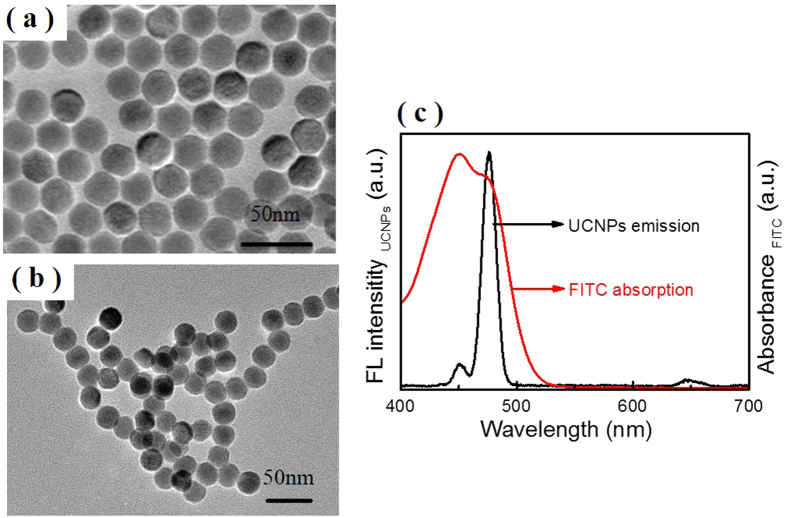
TEM image of (**a**) UCNPs and (**b**) F-UCNPs. (**c**) Emission spectrum of UCNPs under 980 nm excitation (black line) and the absorption spectrum of FITC in water (red line).

To construct the FRET-based self-ratiometric pH nanoprobes, the as-prepared NaYF_4_:Yb^3+^, Tm^3+^ UCNPs were chosen as the donors. As shown in Fig. 2a, UNCPs are well mono-dispersed and uniform with an average size of 21.8 ± 0.7 nm, the high uniformity in size is expected to help improve the reproducibility and precision of the measurement. The diffraction peaks of the UCNPs can be indexed as a pure hexagonal phase of NaYF_4_ (Fig. S2). For FITC conjugation, the oleic acid (OA) coated UCNPs were modified with polyethyleneimine (PEI). The surface modification of UCNPs is monitored by FT-IR spectra, as shown in Fig. S3. After removing the OA ligands, the corresponding peaks of -COO at 1464 cm^−1^, 1565 cm^−1^ and methylene group at 2927 cm^−1^, 2855 cm^−1^ in FT-IR spectrum of OA-UCNPs disappeared, indicating that the OA ligands were successfully removed from the surface of OA-UCNPs. Successful PEI modification of UCNPs was ensured by the appearance of the strong characteristic peaks of free -NH_2_ (1538 cm^−1^), the C-N bond (1397 cm^−1^) and the amine N-H bond (1637 cm^−1^). The content of PEI ligands was also estimated by thermogravimetric analysis (TGA) (Fig. S4). A small weight loss (~ 0.6 *wt*%) below 200 °C is from the absorbed water. The weight loss from 200 °C to 600 °C is ascribed to the decomposition of PEI ligand. It is found that the content of PEI on UCNPs is approximately 2.63 *wt*%.

The emission of PEI-UCNPs is hardly influenced by various pH buffers (<1.6%) (Fig. S5a) and shows a high stability (<3.56%) even after being oscillated for 48 h in different buffers (Fig. S5b), which laid the foundation for accurate self-ratiometric pH sensing. Figure 2c shows the spectra of upconversion luminescence and FITC absorption. The characteristic peaks of NaYF_4_:Yb^3+^, Tm^3+^ UCNPs appear at 450 nm, 475 nm and 645 nm, corresponding to ^1^D_2_ – ^3^F_4_, ^1^G_4_ – ^3^H_6_ and ^1^G_4_ – ^3^F_4_ transitions of Tm^3+^, respectively^35^. It can be seen that there is a large spectral overlap between the absorption of FITC and the 475 nm emission of UCNPs, indicating there appears great probability of FRET between UPNPs and FITC.

### Characterization of F-UCNPs nanoprobe

**Figure 3:**
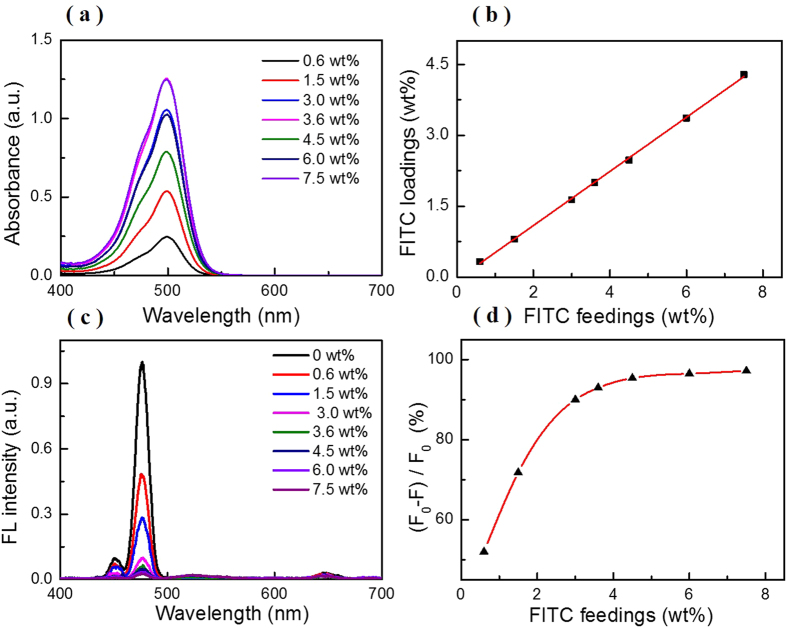
(**a**) Absorption spectra of the supernatant collected after each washing step of FITC conjugating with UCNPs at a series of FITC feedings. The supernatants from 4.5 *wt*% to 7.5 *wt*% were diluted with deionized water. (**b**) Correlation between FITC feedings (*wt*%) and FITC loadings (*wt*%). (**c**) Fluorescent spectra of F-UCNPs with different FITC feeding amounts under excitation of 980 nm and (**d**) Fitting curve of the energy transfer efficiency with FITC feedings, F and F_0_ represent the fluorescence intensity of F-UCNPs and UCNPs, respectively.

**Figure 4:**
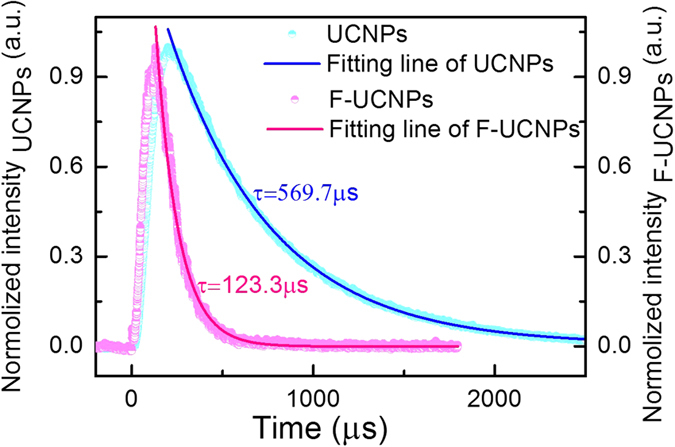
Luminescence decay curves of upconversion emissions monitored at 475 nm and the fitting curves for UCNPs (blue) and FITC-UCNPs (pink).

To have a highly-sensitive pH measurement, it is important to pursue the optimal FITC loading amount. The absorption spectra of the supernatant with FITC feeding amount from 0.6 *wt*% to 7.5 *wt*% are shown in Fig. 3a. The FITC loading amount was calculated by subtracting the amount in the supernatant (unloaded) from the feeding amount, where the unloaded amount was quantified by the absorption spectra of supernatant and the extinction coefficient of FITC (Fig. S6). It can be seen that FITC loadings increased linearly with FITC feedings (Fig. 3b). Figure 3c gives the luminescence spectra of F-UCNPs under 980 nm excitation, the luminescence intensity at 475 nm decreases dramatically with the increase of FITC feeding amount. From the fitting curve (Fig. 3d), it is readily observed that the saturation point appears at 4.5 *wt*%. Therefore, 4.5 *wt*% is adopted as the optimal quantity for F-UCNPs construction (corresponding to 2.5 *wt%* of loading amount, relatively high compared with the typical fluorescence dye loadings in nanosensors ranging from 0.1% to 1%^4^,^8^,^17^,^36^). The F-UCNPs were well-dispersed and uniform (about 22 nm) (Fig. 2b), DLS is shown in Fig. S7. The upconversion luminescence lifetime of UCNPs at 475 nm was measured and fitted to be 569.7 ± 0.6 *μs*, while for F-UCNPs it was shortened to 123.3 ± 0.2 *μs* (Fig. 4). The FRET efficiency of 78.4% is calculated from the formula *E*_*FRET*_ = 1 − *τ*_*DA*_/*τ*_*D*_ where *τ*_*DA*_ and *τ*_*D*_ are the lifetimes of the donor with and without the acceptor, respectively^37^. The high FRET efficiency promoted a high sensitivity of detection.

The stability of FITC on UCNPs surface was studied from the absorption spectra of F-UCNPs subject to oscillation in water. As shown in Fig. S8, as high as 95.9% FITC was retained after being treated for 168 h, demonstrating the robustness of FITC binding with UCNPs. The stability of F-UCNPs in different buffers was verified and the results are shown in Fig. S9. Very small change (<4.6%) was observed in the relative emission ratio (*I/I*_*0*_) after 48 h, where *I*_*0*_ and *I* represent the original emission ratio (I_475_/I_645_) and the emission ratio after 48 h, respectively. The high stability is critical for the accuracy of pH sensing.

### Self-ratiometric pH measurement in buffers

**Figure 5:**
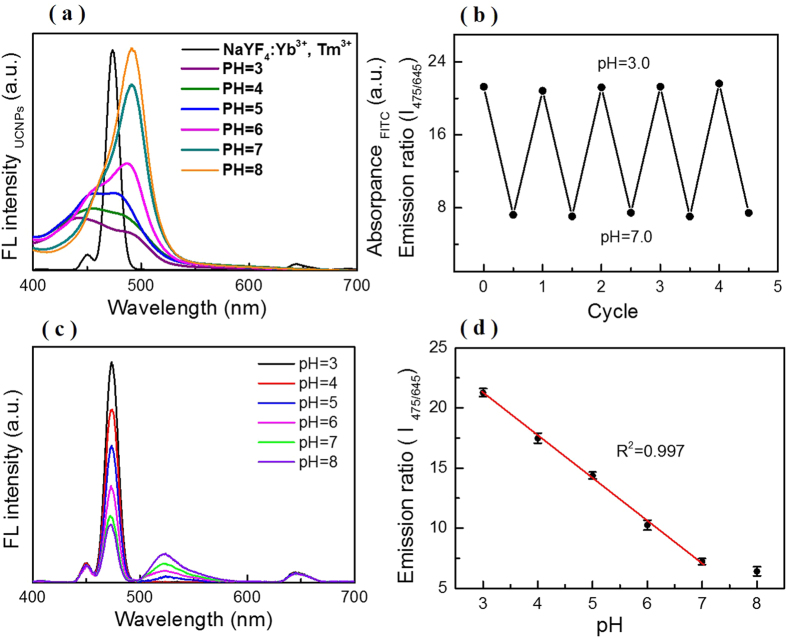
(**a**) Illustration of spectra overlaps between the emission of UCNPs and the absorption of F-UCNPs in different buffers with pH ranging from 3.0 to 8.0. (**b**) The ratio of the relative emission intensity *I*_*475/645*_ (λex = 980 nm) when pH varied with HCl and NaOH solutions from 3.0 to 7.0, repeatedly. (**c**) Luminescence spectra of F-UCNPs with pH value from 3.0 to 8.0 under 980 nm excitation. (**d**) The linear relationship between the ratio *I*_*475/645*_ and pH value. Standard deviations were obtained from four independent experiments.

The feasibility of F-UCNPs nanoprobe in pH sensing is demonstrated in Fig. 5a, where the emission spectra of UCNPs and the absorption spectra of F-UCNPs in different buffers are provided. The spectra overlap exhibits significant change in the range of 3.0–8.0, guaranteeing a high pH sensing sensitivity. Figure 5c displays the upconversion luminescence spectra of F-UCNPs in different pH buffers. *I*_*475*_ shows an obvious decrease with pH increasing, mainly arising from FRET between the UCNPs and FITC. While *I*_*645*_ remains virtually unchanged which is in favor of being used as a reference signal. To determine whether the ratio of the emission relative intensity (*I*_*475/645*_) could be used for quantitative pH measurement, the relation between *I*_*475/645*_ and pH value was plotted in Fig. 5d. Excitingly, there is a good linear relationship in the wide range of 3.0–7.0 which is the typical pH range for cell organelles and pathological cell^38^. The coefficient of correlation (R^2^) is calculated to be 0.997, which shows that the approach has a highly accuracy of pH_i_ detection. F-UCNPs exhibited extremely high detection sensitivity with *I*_*475/650*_ varying 3.63 per unit change in pH value, which was far higher than previous fluorescence-based nanoprobes of 0.3–1.0^3^,^11^,^13^,^29^. The standard deviations are below 0.35 in pH range from 3.0 to 7.0. The high sensitivity and accuracy can be attributed to the efficient energy transfer between UCNPs and FITC, and the lack of auto-fluorescence background under NIR-excitation. To verify the reversibility of F-UCNPs in pH sensing, pH is changed from 3.0 to 7.0 for four cycles. As shown in Fig. 5b, *I*_*475/650*_ shows high stability in four cycles for each pH, demonstrating that F-UCNPs owns splendid reproducibility and reversibility in the pH range.

### Cell cytotoxicity and intracellular imaging of F-UCNPs

As usual, biocompatibility of F-UCNPs nanoprobe is the primary concern in this case. Cell cytotoxicity of F-UCNPs was firstly evaluated using a standard 3-(4,5-dimethythiazol-2-yl)-2,5-diphenyl tetrazolium bromide (MTT) assay. As shown in Fig. S10, the viability of QBC939 cells was up to >80% even when the concentration of F-UCNPs was 800 μg/mL. The result indicated a good biocompatibility of F-UCNPs for pH_i_ sensing.

**Figure 6:**
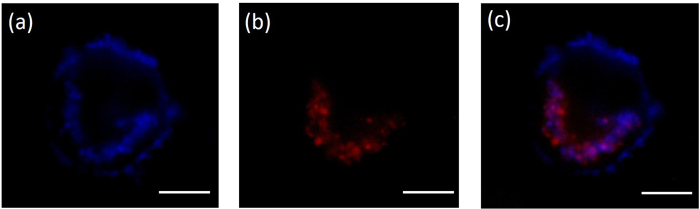
Images of QBC939 cells incubated with F-UCNPs, (**a**) upconversion luminescence image under 980 nm excitation and (**b**) LysoTracker Red image under 532 nm excitation; (**c**) the merged image of (**a**) and (**b**). Scale bar = 10 μm.

The cellular uptake of F-UCNPs into QBC939 cells was evaluated with the aid of confocal microscopy. Before imaging, the cells were washed sufficiently with PBS buffer to remove the free F-UCNPs in solution or on the cell surface. Figure 6a shows the upconversion luminescence image in blue channel (435–485 nm) under 980 nm excitation. Figure 6b shows the image of cells stained by LysoTracker Red (a typical lysosome marker) which was collected in the red channel (600–660 nm) under 532 nm excitation. Co-localization (violet area) of the two images at lysosome was observed, as shown in Fig. 6c, demonstrating the uptake of F-UCNPs by QBC939 cells.

### Intracellular pH calibration and quantitative measurements

**Figure 7:**
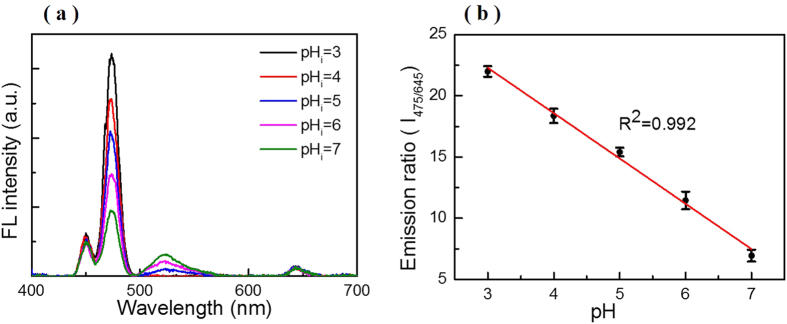
(**a**) Luminescence spectra of QBC939 cells (incubated with F-UCNPs) under 980 nm excitation at different pH, the pH is changed from 3.0 to 7.0. The spectra were normalized at 645 nm. (**b**) The linear relationship of the relative emission intensity ratio (*I*_*475/645*_) versus the pH value. Standard deviations were obtained from four independent experiments.

A direct spectral method was developed based on NIR-excited F-UCNPs nanoprobes for quantitatively determining pH_i_, instead of the traditional software-based analysis (such as MATLAB, Olympus software, *etc.*). As a proof of principle, the intracellular pH value was modulated by incubation in different pH buffers (pH = 3.0–7.0) containing 50 μM nigericin which could homogenize the intracellular and extracellular pH^38^,^39^. Upconversion luminescence spectra of F-UCNPs-incubated cells are shown in Fig. 7a. The negligible background demonstrates the F-UCNPs are fit to be applied in complex environment of cells. Figure 7b is the plot of *I*_*475/650*_ against pH value, which presents a similar linear change with that in pH buffers. The sensing sensitivity is 3.56 per unit change in pH value with the calculated coefficient of correlation (R^2^) being 0.992, which is better than the current reports for pH_i_ detection^3^,^13^,^29^. The sensitivity and coefficient of correlation were only slightly lower than that in buffers, indicating that the complex intracellular micro-environment has little influence on F-UCNPs nanoprobes.

**Figure 8:**
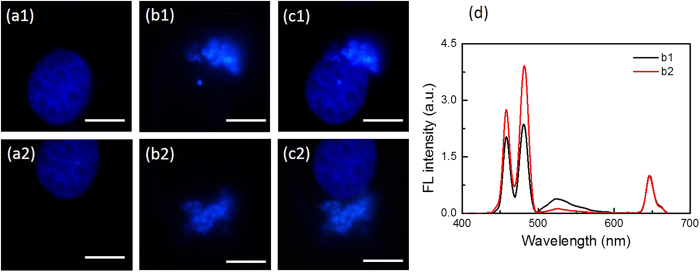
Images and luminescence spectra of QBC939 cells incubated with F-UCNPs, (a1) and (a2) are images of the same cell nucleus (stained by DAPI) under 405 nm excitation. (b1) and (b2) are the upconversion luminescence images of different sites in one cell under 980 nm excitation. (c1) and (c2) are the merged images of (a1) - (b1) and (a2) - (b2), respectively. Scale bar = 10 μm. (**d**) The luminescence spectra of (b1) and (b2) sites of the cell. The spectra were normalized at 645 nm.

Further, to show that the F-UPNPs are capable of detecting local microenvironment of pH_i_ in living cells, the fluorescence confocal imaging and the corresponding spectra measurements of QBC939 cell incubated with the nanoprobes were performed. Figure 8 shows the fluorescence confocal bio-images and spectra of different sites in one cell. The (a1) and (a2) are images of the same cell nucleus (stained by DAPI) which are collected in the blue channel (435–485 nm) under 405 nm excitation. (b1) and (b2) are the upconversion luminescence images of different sites in one cell in the blue channel (435–485 nm) under 980 nm excitation. The (c1) and (c2) are the merged images of (a1) - (b1) and (a2) - (b2), respectively. The corresponding luminescence spectra of (b1) and (b2) are shown in Fig. 8d. According to the linear relationship in Fig. S11, pH_i_ of (b1) and (b2) sites is calculated to be 6.4 and 4.8, which is consistent with the reported pH value of early endosome and lysosome^5^,^13^.

## Conclusion

In conclusion, we have developed a highly-sensitive and accurate quantitative sensing approach of pH_i_ based on self-ratiometric upconversion nanoprobes, which circumvents the drawbacks of the current fluorescence-based approaches. The F-UCNPs nanoprobe exhibits high FITC loading amounts, excellent structural stability, high energy transfer efficiency, low cell cytotoxicity and excellent pH_i_ quantitative sensing property. A highly sensitive sensing of 3.56 per unit change in pH_i_ sensing from 3.0 to 7.0 and an accurate quantitative detection of pH_i_ with deviation less than 0.43 have been realized. This novel approach shall facilitate greatly the cellular local microenvironment studies, as well as designing intracellular drug delivery systems.

## Methods

### Construction of self-ratiometric UCNPs-based pH nanoprobe

The FRET-based self-ratiometric pH nanoprobe was constructed by conjugating pH-sensitive FITC (acceptor) onto the surface of polyethyleneimine-modified UCNPs (PEI-UCNPs) (donor). The ethanol solution of FITC (1 mg/mL) was added into PEI-UCNPs of 1 mg in deionized water of 1 mL, different FITC feeding amounts (0.6 *wt*%, 1.5 *wt*%, 3.0 *wt*%, 3.6 *wt*%, 4.5 *wt*%, 6.0 *wt*%, 7.5 *wt*%) were used to evaluate the correlation between FITC feedings and the quenching efficiency of upconversion emission at 475 nm. The mixture was stirred mildly for 24 h in the dark environment to obtain FITC-conjugated UCNPs (F-UCNPs), then was centrifuged (9000 r/min, 4 °C, 15 min) for three times and re-dissolved in deionized water of 1 mL. The absorption spectra of the collected supernatants (2 mL for 0.6–3.6 *wt*% and 4 mL for 4.5–7.5 *wt*%) were measured, respectively. The emission spectra of F-UCNPs under 980 nm excitation were measured via a Hitachi F-4500 fluorescence spectrofluorimeter. FITC was dissolved in deionized water to obtain a series of FITC solutions, the absorption spectra were recorded and a standard curve of FITC concentration dependent absorbance was plotted. The FITC loading amount of F-UCNPs was calculated by subtracting the amount in the supernatant from the feeding amount.

To evaluate the stability of FITC on UCNPs surface, F-UCNPs aqueous solutions of 0.5 mg/mL were oscillated with different time (0 h, 5 h, 10 h, 24 h, 48 h, 96 h and 168 h, respectively) and centrifuged at 9000 r/min for 15 min. FITC residual amount was determined from the absorption spectra. For pH measurement, the stability of F-UCNPs in different buffers (pH = 3.0–8.0) were firstly evaluated. F-UCNPs buffer solutions of 0.5 mg/mL were oscillated for 48 h and centrifuged. Luminescence spectra at 0 h and 48 h were measured, respectively.

### pH measurement in different buffers

The pH response of F-UCNPs nanoprobes was evaluated by measuring the change of upconversion luminescence intensity of F-UNCPs. F-UCNPs of 1 mg was dispersed in buffers of 2 mL, where these buffers were prepared for pH 3.0, 4.0, 5.0 and 6.0 (3.0–6.0) with citrate buffers, and for pH 7.0 and 8.0 (7.0–8.0) with phosphate ones, respectively. The absorption and luminescence spectra in buffers with different pH above (3.0–8.0) were measured, respectively. The self-ratiometric measurement was achieved by comparing the luminescence intensity at 475 nm (*I*_475_) with that one at 645 nm (*I*_645_). *I*_475_ depended on pH, while *I*_645_ was inert to pH due to no spectral overlap between the absorption of FITC and emission at 645 nm of UCNPs. The corresponding pH calibration curve was obtained by plotting *I*_*475/645*_ versus pH value. To ensure the accuracy, the standard deviations were shown from four independent experiments.

To evaluate the reversibility of pH sensing, F-UCNPs of 0.5 mg/mL were dispersed in citrate buffered solution (pH = 3.0), and the pH of the solution was changed from 3.0 to 7.0 and vice versa for four cycles using 1 M HCl and 1 M NaOH solutions. Upconversion luminescence spectra at each pH value were measured.

### Cell cytotoxicity of F-UCNPs nanoprobes

QBC939 cells were cultured in RPMI-1640 medium with 10% fetal bovine serum, 100 IU/mL penicillin and 100 μg/mL streptomycin. The cell cytotoxicity was assessed by a standard MTT assay^25^. Briefly, QBC939 cells were seeded in a 96-well plate at a density of 8000–10000 cells per well and incubated for 24 h at 37 °C under 5% CO_2_. Then, 100 μL of F-UCNPs solution were added to the culture medium with different concentrations (0, 25, 50, 100, 200, 400, 800 μg/mL), each concentration set five parallel wells. After being incubated for 24 h, the viabilities of cells were estimated by standard MTT assay.

### Intracellular Imaging of F-UCNPs nanoprobes

QBC939 cells were seeded on a glass-bottom petri dish at a density of 1.5 × 10^4^ cells per dish and cultured for 24 h. 100 μL of F-UCNPs solution (1 mg/mL) was added into the dish. After 12 h incubation, the dish was washed for four times with 10 mM PBS. The cells were then stained with 0.1 mg/mL LysoTracker Red for 30 min and washed for three times with PBS again. The cell imaging was collected using a Nikon Inverted Microscope Eclipse Ti-U Main Body (Nikon, Tokyo, Japan) equipped with C2-SHS Scanner and Controller. The image of UCNPs was collected in blue channel (435–485 nm) under excitation of a diode laser with 980 nm. The image of LysoTracker Red was collected in red channel (600–660 nm) under excitation of a laser with 532 nm.

### Intracellular pH calibration

QBC939 cells were seeded in a 6-well plate at a density of 5 × 10^4^ cells per well and incubated for 24 h at 37 °C under 5% CO_2_. Each well of the plate was added with 500 μL of F-UCNPs (1 mg/mL) and followed by another 12 h of incubation. After being washed for three times with PBS, the cells were digested from culture dish and centrifuged at 1200 r/min for 4 min. The cells from each well were re-suspended in 2 mL solutions (120 mM KCl, 1 mM CaCl_2_, 0.5 mM MgSO_4_, 10 mM NaCl, 10 mM different pH buffers) of pH ranging from 3.0 to 7.0, containing 50 μM nigericin, respectively. After the cells were incubated in the solution for 15 min, the luminescence spectra of the above solutions were recorded by a Hitachi F-4500 fluorescence spectrofluorimeter under excitation of 980 nm, respectively. And the corresponding pH_i_ calibration curve was obtained directly.

### Fluorescence imaging and direct spectra measurements of cells incubated with F-UCNPs

To validate the ability of F-UCNPs for the quantitative measurement of pH_i_, the cells incubated with F-UCNPs were firstly fixed in 4% paraformaldehyde for 15 min, and then were washed for three times with PBS. The nucleus were stained with DAPI of 0.1 μg/mL for 5 min, then washed for three times with PBS again. The different sites in one cell were imaged, and the corresponding luminescence spectra were recorded, respectively. The images of UCNPs and DAPI were collected in blue channel (435–485 nm) under 980 nm and 405 nm excitation, respectively. The luminescence spectra of F-UCNPs in the cell and different buffers were collected by a Maya 2000 visible spectrometer (Ocean optics) under 980 nm excitation.

## Additional Information

**How to cite this article**: Li, C. *et al*. Accurate Quantitative Sensing of Intracellular pH based on Self-ratiometric Upconversion Luminescent Nanoprobe. *Sci. Rep.* **6**, 38617; doi: 10.1038/srep38617 (2016).

**Publisher's note:** Springer Nature remains neutral with regard to jurisdictional claims in published maps and institutional affiliations.

## Supplementary Material

Supplementary Information
