# Supplementary material for: Accurate Quantitative Sensing of Intracellular pH based on Self-ratiometric Upconversion Luminescent Nanoprobe
Source: Sci Rep. 2016 Dec 9;6:38617. doi: 10.1038/srep38617 (PMC5146920; doi:10.1038/srep38617)
Supplement: Supplementary Information [file srep38617-s1.pdf]

Supplementary Information:

Accurate Quantitative Sensing of Intracellular pH based on Self-ratiometric Upconversion Luminescent Nanoprobe

Cuixia Li,[1,2] Jing Zuo,[1,2,4] Li Zhang,[3] Yulei Chang,[1] Youlin Zhang,*[1] Langping Tu,[1,4] Xiaomin Liu,[1] Bin Xue,[1,4] Qiqing Li,[1,2,4] Huiying Zhao,[3] Hong Zhang*[4] and Xianggui Kong,*[1]

[1] State Key Laboratory of Luminescence and Applications, Changchun Institute of Optics, Fine Mechanics and Physics, Chinese Academy of Sciences, Changchun 130033, China. E-mail: *xgkong14@ciomp.ac.cn; zhangyl0109@sina.com*

[2] Graduate University of the Chinese Academy of Sciences, Beijing 100049, China

[3] Department of Basic Medicine, Gerontology Department of First Bethune Hospital, University of Jilin, Changchun 130021, China.

[4] Van't Hoff Institute for Molecular Sciences, University of Amsterdam, Science Park 904, 1098 XH Amsterdam, The Netherlands. E-mail: *h.zhang@uva.nl*

Correspondence to *xgkong14@ciomp.ac.cn*; *zhangyl0109@sina.com; h.zhang@uva.nl*.

Materials and methods

Materials

FITC, Citric acid monohydrate, Trisodium citrate dihydrate, Sodium dihydrogen phosphate dehydrate and Disodium hydrogen phosphate dodecahydrate were purchased from Aladdin. LysoTracker Red and DAPI was purchased from Invitrogen. The other chemicals were purchased from Sigma-Aldrich. Polyethyleneimine (PEI) was branched (MW~1800) and the structural formula was shown in Fig. S1. All the materials were of analytical or chemical pure grade and were used without further purification. The aqueous solutions were prepared using deionized water (Mill-Q, Millipore, 18.2 MΩ resistivity). QBC939 cells (Human Cholangiocarcinoma Cell Line) were purchased from First Bethune Hospital, University of Jilin. The cultured medium RPMI1640, fetal bovine serum and Trypsin-EDTA Solution were purchased from Dingguo Biotechnology Development (China).

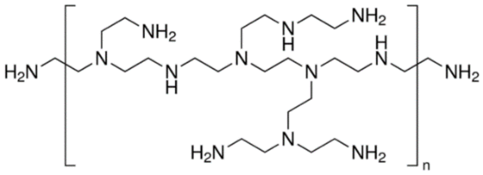

Figure. S1 the structural formula of branched PEI.

Apparatus

The size and morphology of the prepared UCNPs were characterized by a JEM-2100F electron microscope operated at 200 kV. The crystal structure of UCNPs was determined by a Bruker D8-advance X-ray diffractometer (XRD) with Cu Kα irradiation (λ=1.5418 Å) and 2θ range from 10° to 80°. FT-IR spectra of UCNPs were acquired using VERTEX 70 FT-IR spectrometer with the KBr technique. The UV-Vis absorption spectra were detected using a UV-3101 spectrophotometer. The upconversion emission spectra were measured by a Hitachi F-4500 fluorescence spectrofluorimeter and a Maya 2000 visible spectrometer (Ocean optics) equipped with a 980 nm diode CW laser as the excitation source. Upconversion luminescence

kinetics was measured by a 500 MHz Tektronix digital oscilloscope with the excitation of a nanosecond pulse train at 980 nm from an optical parametric oscillator. pH of the buffers was calibrated with a pH meter (Sartorius PB-10). The thermogravimetric analysis (TGA) curve was recorded with a thermal analysis instrument (Perkine Elmer Pyris 1) with a heating rate of 10 °C/min in a nitrogen flow of 100 mL/min.

Synthesis and amino-modification of $NaYF_4$:Yb^{3+},Tm^{3+} UCNPs

$NaYF_4$:24.7%Yb,0.3%Tm UCNPs were synthesized according to previous report and were dispersed in cyclohexane with oleic acid ligands on the surface.[1] In order to conjugate FITC, UCNPs were firstly modified with PEI for amino-terminal.[2] Briefly, 8 mL of 10 mg/mL UCNPs solution in cyclohexane mixed with 8 mL of 0.1 M HCl solution. The mixture was stirred overnight to obtain ligand-free UCNPs. After centrifugation (11500 r/min, 4 °C, 20 min) twice, the ligand-free UCNPs were re-dispersed in 18 mL deionized water. 170 mg PEI (50 *wt*%) was added into the above solution and stirred mildly for 24 h to obtain PEI-modified UCNPs (PEI-UCNPs). After centrifugation (11500 r/min, 4 °C, 15 min) for three times, PEI-UCNPs were dispersed in 8 mL water. To evaluate the emission stability of PEI-UCNPs, PEI-UCNPs in varied pH buffers (pH=3.0-8.0) were tuned to the same concentration (0.5 mg/mL), the luminescence spectra were measured under 980 nm excitation, respectively. Then, the above solutions were oscillated for 48 h and the luminescence spectra were measured again.

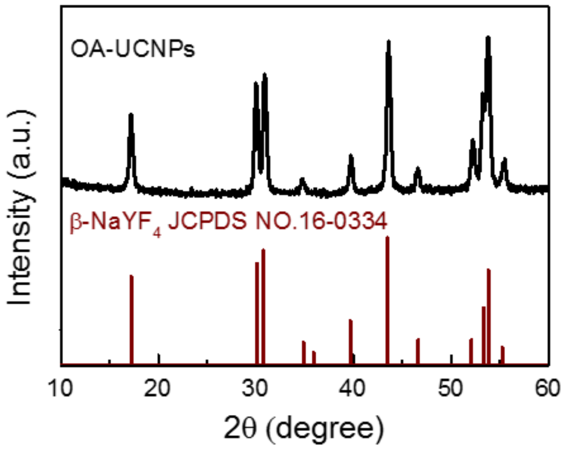

Figure. S2 XRD pattern of $NaYF_4:Yb^{3+},Tm^{3+}$ UCNPs (black line) and the standard line pattern of β-NaYF4 (red line).

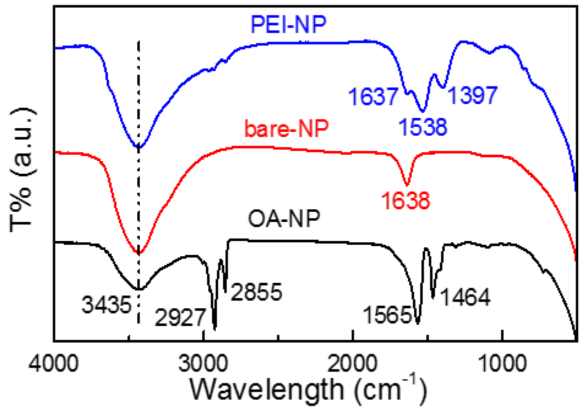

Figure. S3 FT-IR spectra of OA-UCNPs (black line), ligand-free UCNPs (red line) and PEI-UCNPs (blue line). The broad peak at 3435cm^{-1} was attributed to the -OH vibration of the adsorbed water.

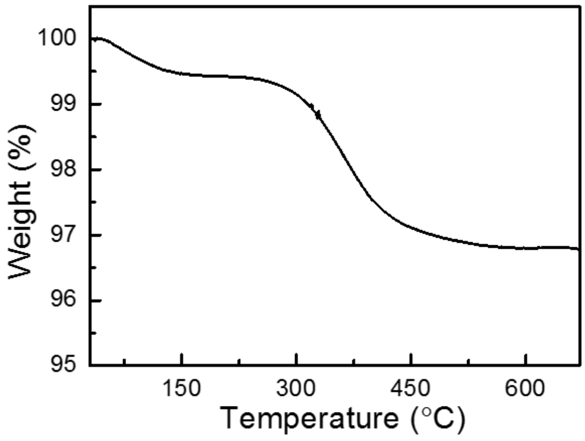

Figure. S4 TGA curves of the as-prepared PEI-UCNPs.

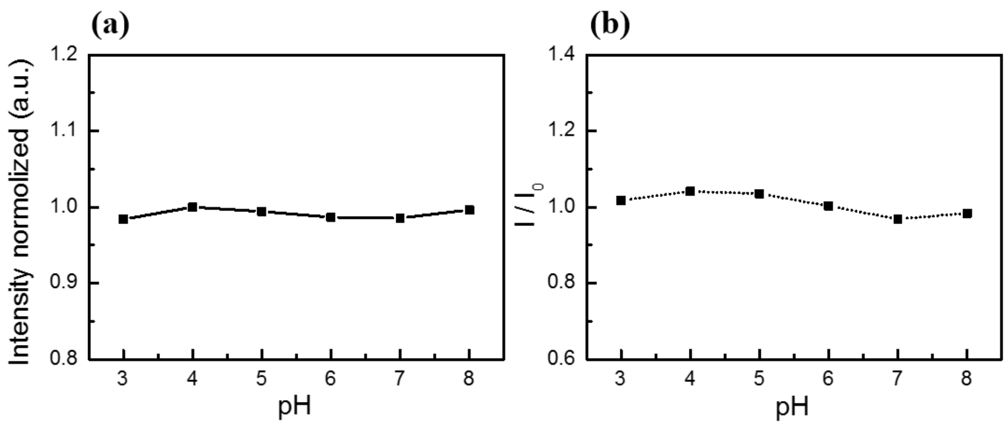

Figure. S5 Emission stability of PEI-UCNPs. (a) Luminescence intensity of PEI-UCNPs in varied pH buffers, the data was normalized at pH=4. (b) Emission ratio of PEI-UCNPs after being oscillated for 48 h in different pH buffers, I_0 and I represent the luminescence intensity of PEI-UCNPs at 0 h and 48 h, respectively.

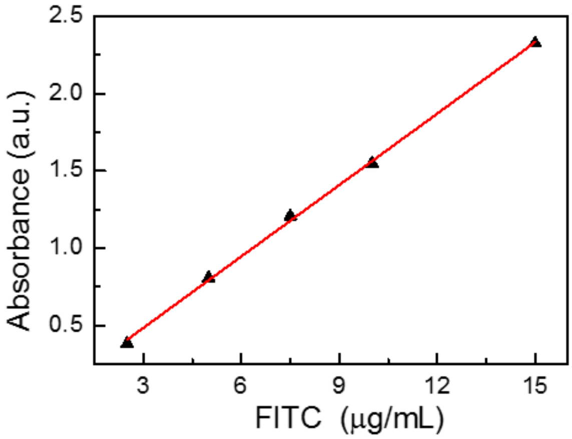

Figure. S6 Standard curve of FITC in PEI-UCNPs water solution versus FITC concentration by UV-Vis.

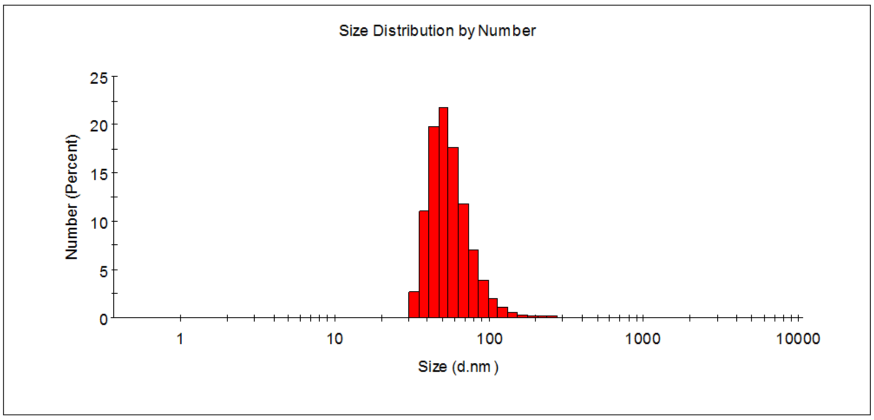

Figure. S7 Number weighted dynamic light scattering measurements of F-UCNPs in water.

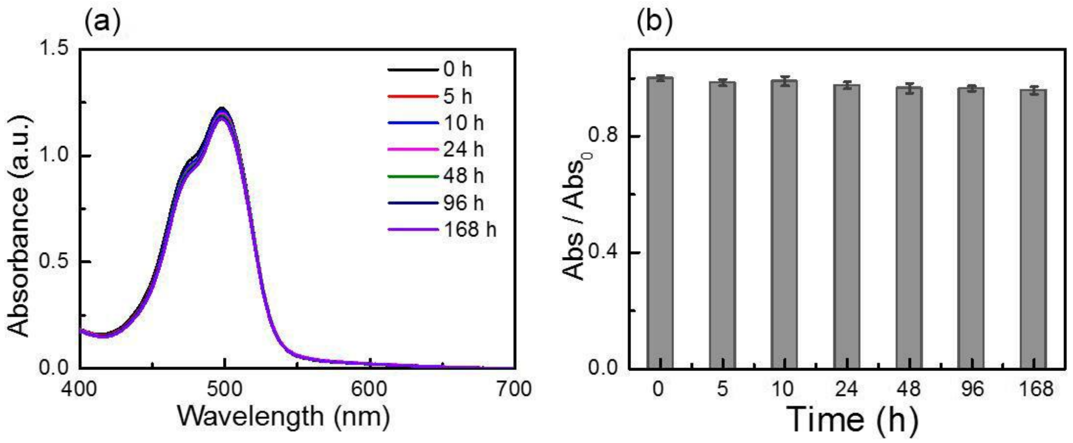

Figure. S8 Stability of prepared F-UCNPs nanoprobe. (a) The absorption spectra of F-UCNPs being oscillated in water for 0 - 168 h, respectively. (b) The surplus ratio of FITC after being oscillated for different time. Abs_0 and Abs represented the absorbance of F-UCNPs after being oscillated for 0 h and 5 - 168 h, respectively.

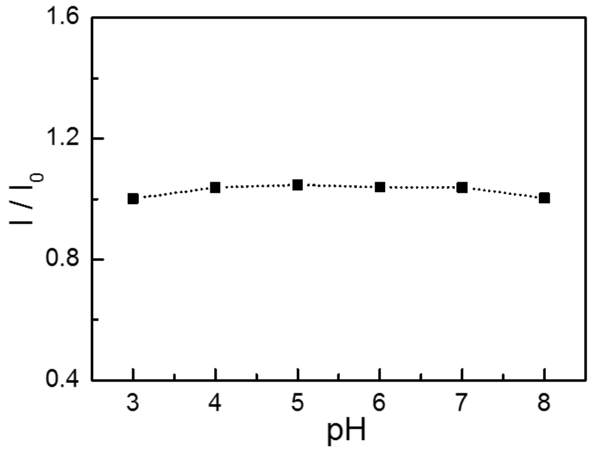

Figure. S9 The relative emission ratio (I/I_0) of F-UCNPs in different buffers (pH from 3.0 to 8.0) after 48 h. I_0 and I represent the original emission ratio (I_{475}/I_{645}) and the emission ratio after 48 h, respectively.

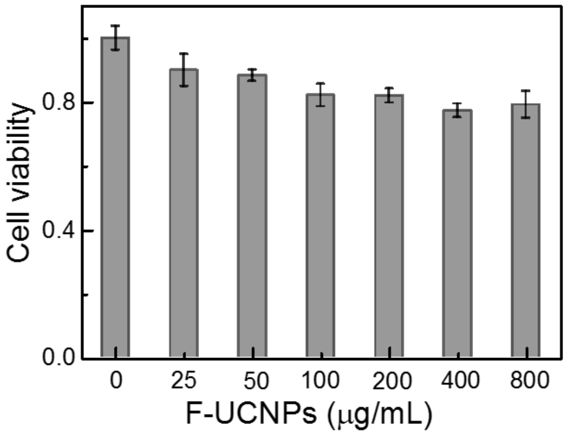

Figure. S10 Cell viability obtained by MTT experiments. QBC939 cells were incubated with a series of concentration of F-UCNPs for 24 h.

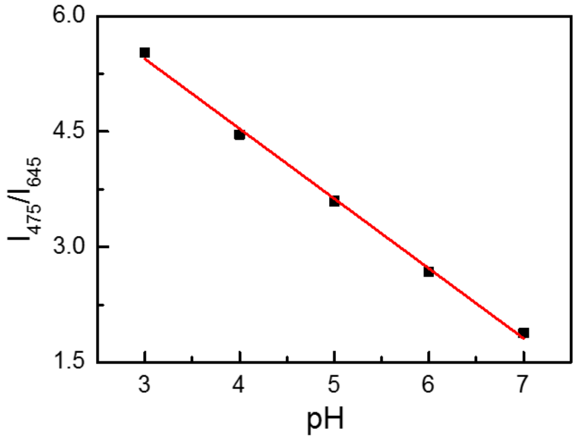

Figure. S11 Linear relationship of the relative emission intensity ratio ($I_{475/645}$) versus the pH value under confocal microscopy measurement.

References

1. Li, Z. & Zhang, Y. *Nanotechnology* **19**, 345606 (2008).

2. Bogdan, N., Vetrone, F., Ozin, G. A. & Capobianco, J. A. Synthesis of ligand-free colloidally stable water dispersible brightly luminescent lanthanide-doped upconverting nanoparticles. *Nano Lett* **11,** 835-840 (2011).
